# Primary Motor Cortex Neurons during Individuated Finger and Wrist Movements: Correlation of Spike Firing Rates with the Motion of Individual Digits versus Their Principal Components

**DOI:** 10.3389/fneur.2014.00070

**Published:** 2014-05-19

**Authors:** Evan Kirsch, Gil Rivlis, Marc H. Schieber

**Affiliations:** ^1^Department of Biomedical Engineering, University of Rochester, Rochester, NY, USA; ^2^Department of Neurology, University of Rochester, Rochester, NY, USA; ^3^Department of Neurobiology and Anatomy, University of Rochester, Rochester, NY, USA

**Keywords:** cortico-motoneuronal, electromyography, hand, joint angle, kinematic synergy, principal component, spike-triggered average

## Abstract

The joints of the hand provide 24 mechanical degrees of freedom. Yet 2–7 principal components (PCs) account for 80–95% of the variance in hand joint motion during tasks that vary from grasping to finger spelling. Such findings have led to the hypothesis that the brain may simplify operation of the hand by preferentially controlling PCs. We tested this hypothesis using data recorded from the primary motor cortex (M1) during individuated finger and wrist movements. Principal component analysis (PCA) of the simultaneous position of the five digits and the wrist showed relatively consistent kinematic synergies across recording sessions in two monkeys. The first three PCs typically accounted for 85% of the variance. Cross-correlations then were calculated between the firing rate of single neurons and the simultaneous flexion/extension motion of each of the five digits and the wrist, as well as with each of their six PCs. For each neuron, we then compared the maximal absolute value of the cross-correlations (MAXC) achieved with the motion of any digit or the wrist to the MAXC achieved with motion along any PC axis. The MAXC with a digit and the MAXC with a PC were themselves highly correlated across neurons. A minority of neurons correlated more strongly with a PC than with any digit. But for the populations of neurons sampled from each of two subjects, MAXCs with digits were slightly but significantly higher than those with PCs. We therefore reject the hypothesis that M1 neurons preferentially control PCs of hand motion. We cannot exclude the possibility that M1 neurons might control kinematic synergies identified using linear or non-linear methods other than PCA. We consider it more likely, however, that neurons in other centers of the motor system – such as the pontomedullary reticular formation and the spinal gray matter – drive synergies of movement and/or muscles, which M1 neurons act to fractionate in producing individuated finger and wrist movements.

## Introduction

The digits of the hand commonly have been thought to move independently of one another. But kinematic analysis has shown that simultaneous motion of multiple fingers occurs in virtually all human hand and finger movements. These include not only activities of daily living such as grasping and haptic exploration (1–4), but also sophisticated performances including finger spelling, typing, or piano playing (5–7), and even the individuated movements made when normal human subjects are asked to move only one finger (8, 9).

When simultaneous variation occurs in many independent elements – whether joint angles, muscles, or neurons – a limited variety of fixed patterns, or synergies, potentially can account for much of the simultaneous variation. The concept of synergies is useful, simplifying the problem of controlling all the original elements, primarily if the number of synergies needed to account for most of the variation in the data is substantially less than the number of original elements. Several different mathematical approaches, both linear and non-linear, might be used to identify such synergies, and which approach is most likely to capture synergies potentially used by the nervous system cannot be predicted.

Almost all prior studies of the kinematic synergies involved in hand movements have used a comparatively straightforward, linear approach – principal component analysis (PCA) (10). In the human studies cited above, application of PCA has identified patterns of correlated motion among multiple joints of the fingers and wrist. In general, a small number of such patterns, captured as principal components (PCs), accounts for the vast majority of the variance in the larger number of original elements, here mechanical degrees of freedom (DoFs), typically the rotation of individual joints. Similarly in the grasping movements of non-human primates, the simultaneous correlated motion of multiple DoFs in the thumb, fingers, and wrist can be attributed largely to a small number of PCs (11–15).

These observations have led to the hypothesis that, at some level, the central nervous system (CNS) may simplify the computational burden of controlling the hand by driving PCs of hand kinematics. Patterns of simultaneous correlated movement kinematics, isometric forces, or muscle activity have been attributed variously to the spinal gray matter (16), the pontomedullary reticular formation (PMRF) (17–19), and the motor cortex (20, 21). If the PCs of hand and finger movements are controlled at some level of the CNS, then downstream neural, muscular, or mechanical elements would be responsible for distributing motion to multiple mechanical DoFs simultaneously. Upstream levels of the CNS then also might work in terms of PCs. Alternatively, some upstream centers might bypass the levels driving PCs and superimpose additional control on hand kinematics. Here we examined the PCs of individuated finger and wrist movements in non-human primates, as well as the extent to which neurons in the primary motor cortex (M1) are correlated with these PCs as compared to the original kinematics.

## Materials and Methods

Many of the methods used in the present study for behavioral training, data collection, and initial analyses have been described in previous reports, and are summarized here as needed.

### Animals and behavioral procedures

**Figure 1:**
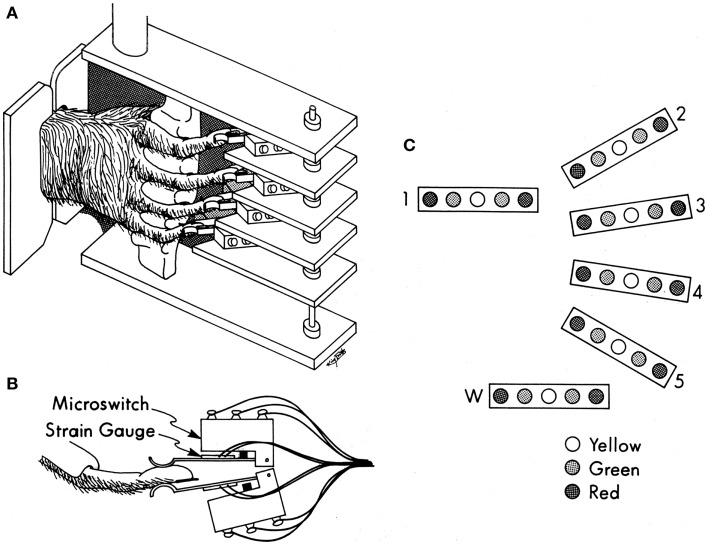
**Manipulandum and display for the individuated finger and wrist movement task**. **(A)** Pistol-grip manipulandum. **(B)** Overhead view of a monkey’s finger between two microswitches with semiconductor strain gages mounted on the lever-arm of each microswitch. **(C)** Display of LEDs used to instruct movements (red) and to inform the monkey for which fingers both switches were open (yellow), or if not, then which one was closed (green). Reproduced with permission from Ref. (22).

All care and use of these purpose-bred monkeys complied with the U.S.P.H.S. Policy on Humane Care and Use of Laboratory Animals, and was approved by the University Committee on Animal Resources at the University of Rochester. Each monkey was trained to perform visually cued individuated flexion and extension movements of the right hand fingers and/or wrist (22). As the monkey sat in a primate chair, the right elbow was held in a molded cast, and the right hand was placed in a pistol-grip manipulandum, which separated each finger into a different slot (Figure 1A). At the end of each slot, the fingertip lay between two microswitches (Figure 1B). By flexing or extending the digit a few millimeters, the monkey closed the ventral or dorsal switch, respectively. The manipulandum, in turn, was mounted on an axis that permitted flexion and extension wrist movements, transduced with a co-axial precision potentiometer. Each monkey viewed a display (Figure 1C) on which each digit (and the wrist) was represented by a row of five light-emitting diodes (LEDs). When the monkey flexed or extended a digit, closing a microswitch, the central yellow LED went out and a green LED to the left or right, respectively, came on, cueing the monkey as to which switch(es) had been closed. For the wrist, the voltage read from the potentiometer crossed fixed levels that substituted for flexion and extension microswitches. Red LEDs to the far left or right were illuminated one at a time, instructing the monkey to close that one switch (or move the wrist). If the monkey closed the instructed switch within the 700 ms response time allowed after illumination of the red instruction LED, and held it closed for a 500 ms final hold period without closing any other switches, the monkey received a water reward. After each rewarded trial, the movement to be instructed for the next trial was rotated in a pseudorandom order. We abbreviate each instructed movement with the number of the instructed digit (1 = thumb through 5 = little finger, 6 or w = wrist), and the first letter of the instructed direction (f – flexion; e – extension), for example, “4f” indicates instructed flexion of the ring finger. The behavioral task was controlled by custom software written in TEMPO (Reflective Computing, Olympia, WA, USA), which also generated 8-bit behavioral event marker codes.

While behavioral performance depended only on the closing of the microswitches for the fingers and the level crossings for the wrist, a continuous analog signal representing the flexion/extension position of each digit was generated using a semiconductor strain gage (BLH SPB3-20-35) mounted on the lever-arm of each microswitch (22). The gages mounted on the flexion and extension switches for each digit were configured as two legs of a Wheatstone bridge, the output of which was amplified, low-pass filtered (5 kHz cutoff), and biased with a commercial circuit (Analog Devices 2B31J). Although the spring qualities of the microswitches and their lever-arms produced a linear relationship between fingertip position and force, here we will consider these signals to represent fingertip position. A separate analog signal representing the flexion/extension position of the wrist was provided by the potentiometer coupled to the wrist axis.

### Data collection

After training, aseptic surgery under isoflurane anesthesia was used to open a craniotomy over the left central sulcus at the level of the hand representation, and to implant both a rectangular Lucite recording chamber over the craniotomy and two head-holding posts. Once the monkey had recovered from this procedure and had become accustomed to performing the finger movement task with its head held stationary, EMG electrodes made of 32 gage, Teflon-insulated, multi-stranded stainless steel wire (Cooner AS632, Chatsworth, CA, USA) were implanted percutaneously using aseptic technique in 8–16 forearm and hand muscles under Ketamine anesthesia, using techniques adapted from those of Cheney and colleagues (23–25). Muscles implanted typically included 8–16 of the following: thenar eminence (Thenar); first dorsal interosseus (FDI); hypothenar eminence (Hypoth); flexor digitorum profundus, radial region (FDPr); flexor digitorum profundus, ulnar region (FDPu); flexor digitorum profundus, proximal ulnar region (FDPpu); flexor digitorum superficialis (FDS); flexor carpi radialis (FCR); palmaris longus (PL); flexor carpi ulnaris (FCU); abductor pollicis longus (APL); extensor pollicis longus (EPL); extensor digiti secundi et tertii (ED23); extensor digitorum communis (EDC); extensor digiti quarti et quinti (ED45); extensor carpi radialis (ECR); extensor carpi ulnaris (ECU), and supinator (Sup).

Thereafter in daily recording sessions, conventional techniques were used to record a single M1 neurons simultaneously with the analog signals representing the flexion/extension position of each digit and the wrist (sampled at 1 kHz) and with EMG activity from the implanted forearm and hand muscles (EMG amplification 2,000–100,000×, bandpass 0.3–3 kHz, sampling frequency ~4 kHz per channel) as the monkey performed individuated finger and wrist movements. During each recording session, two data acquisition interfaces were used to store data to disk on two host PCs, which also provided scrolling displays of all neuron, kinematic and EMG recordings (Power1401 interface, Spike2 software, Cambridge Electronic Design, UK). The same neuron data and behavioral event marker codes were stored in parallel in these two data streams, while the six kinematic signals were stored together on one system along with four EMG channels, and the remaining EMGs were stored on the other system. A third data acquisition interface and host PC running AVE software (courtesy Shupe, Fetz, and Cheney) were used concurrently to form initial on-line averages of rectified EMG for each channel using data segments extending ±50 ms from the time of all neuron spikes.

### Data analysis

#### Principal component analysis

If we consider each original element (here the motion of each of the five digits and of the wrist) as a dimension in an abstract Euclidean space with orthogonal axes, we can consider our data (here the simultaneous positions of the five digits and the wrist at each time step) as a cloud of points in the six-dimensional space. If some of the original elements are correlated, then there will be a direction in this space that accounts for their simultaneous, correlated variation. PCA can be thought of as a translation of the origin and a rotation of the orthogonal axes such that as much of variance in the data points as possible lies along a single axis, which then is defined as that of the first PC (PC1) (10). A unit vector that points in the direction of this new axis is termed the eigenvector of PC1. A second orthogonal axis (PC2) will be found that accounts for as much of the remaining variance as possible, and so forth for as many PCs as there are original dimensions. The orthogonal PC axes thus are another orthogonal coordinate system (a basis) for viewing the same data. Just as a single data point can be considered to have a projection on each of the original axes, so the same data point can be considered to have a projection on each of the PC axes (in the direction of each of the eigenvectors). And as successive points progress in a time series, their projections on both the original axes and on the PC axes progress as time series.

Two important differences exist, however, between the original axes and the PC axes: first, whereas projections of the data along the original dimensions may be correlated, projections of the data in the directions of the PC eigenvectors are uncorrelated. And second, whereas the original elements may each have any amount of variance, the PCs are rank-ordered according to the fraction of the total variance accounted for by each, with PC1 accounting for the most variance and progressively higher-order PCs accounting for progressively less variance. For purposes of identifying synergies and thereby reducing dimensions, low-order PCs are most likely to represent meaningful synergies while high-order PCs that account for little variance can be considered to be “noise” and disregarded.

For the present study, the kinematic data representing the flexion/extension position of each digit and of the wrist was normalized from −1 (greatest extension achieved by that digit) to +1 (greatest flexion achieved by that digit) across each recording session, and downsampled to 200 Hz. PCA performed on these normalized, six-dimensional kinematic data from each recording session then resulted in six PC eigenvectors (the translated and rotated basis of orthonormal unit vectors) rank-ordered according to the variance accounted for by each, and the temporal weighting of each eigenvector as a function of time throughout the recording session.

#### Cross-correlation of neuron firing rate with kinematic variables

To enable cross-correlation of neuron firing rate with kinematic variables, each neuron’s spike train was converted to an analog representation of firing rate as a function of time as:

$$y\left(t\right)=\left\{\begin{array}{cc} {\left(t_{\text{n}-1}-t_{\text{n}-2}\right)}^{-1},\; & t-t_{\text{n}-1}<t_{\text{n}-1}-t_{\text{n}-2}\\ {\left(t-t_{\text{n}-1}\right)}^{-1},\; & t-t_{\text{n}-1}\geq t_{\text{n}-1}-t_{\text{n}-2} \end{array}\right.$$

where *y(t)* is the estimate of the instantaneous firing rate at time *t, t*_n − 1_ is the time of the most recent spike preceding time *t*, and *t*_n − 2_ is the time of the spike preceding *t*_n − 1_. Hence at each 5 ms time step, *t*, the time elapsed since the most recent spike, *t* − *t*_n − 1_, was compared to the interval between the two most recent spikes, *t*_n − 2_ − *t*_n − 1_. If the time elapsed was less than the most recent inter-spike interval, then the instantaneous frequency was set to the inverse of this interval. If the time elapsed was greater than or equal to the most recent inter-spike interval, then the instantaneous frequency was set to the inverse of the interval between the most recent spike and the current time, providing a gradual decay of instantaneous frequency until the occurrence of the next spike.

We then performed cross-correlation of each neuron’s instantaneous firing rate against each of the kinematic variables – both the six original digit and wrist positions and their six PCs – for leads and lags up to ±500 ms. Prior to cross-correlation, each signal was mean-zeroed and normalized such that the auto-covariance at zero lag was 1. Each cross-correlation was performed using data over the entire duration of the recording, which in monkey C averaged 777 ± 228 s (mean ± SD; range: 370–1550 s) and in monkey G averaged 690 ± 273 s (range: 178–1515 s).

## Results

The present data include 49 single-neuron recording sessions made during 38 daily microelectrode penetrations in monkey C, and 155 single-neuron recording sessions made during 83 microelectrode penetrations in monkey G (24, 25).

### Principal components of individuated finger and wrist movements

**Figure 2:**
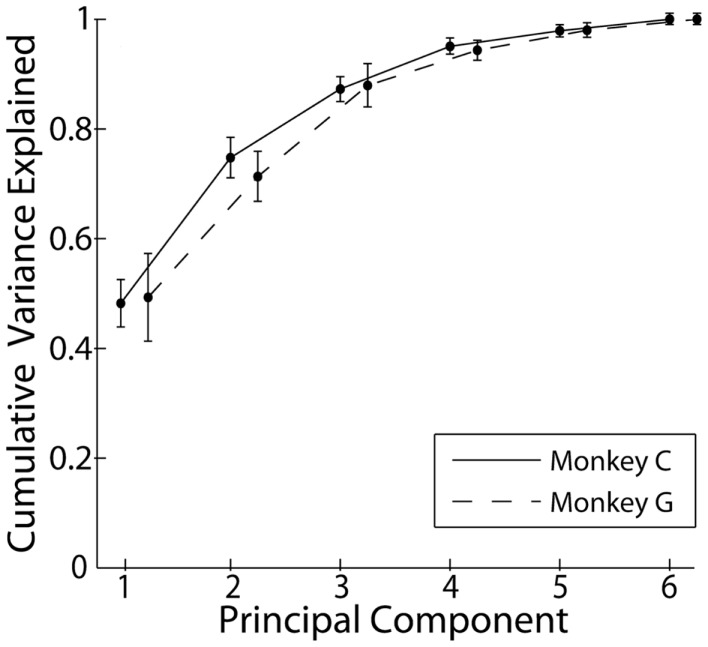
**Cumulative variance explained by the rank-ordered principal components**. Each point represents the mean across all recordings each monkey. Error bars indicate 1 SD.

Principal component analysis was performed on the kinematic data from each recording separately. Figure 2 shows the cumulative variance accounted for as the number of PCs included in rank order increased from 1 to 6. Each point here represents the mean across all sessions from a given monkey. In both monkeys, PC1 accounted for approximately 50% of the variance, and the first three PCs together accounted for approximately 85% of the variance. Consistent with other studies that have applied PCA to the hand movements of both humans and non-human primates, a few low-order PCs thus accounted for the large majority of the variance in the present individuated finger and wrist movements.

**Figure 3:**
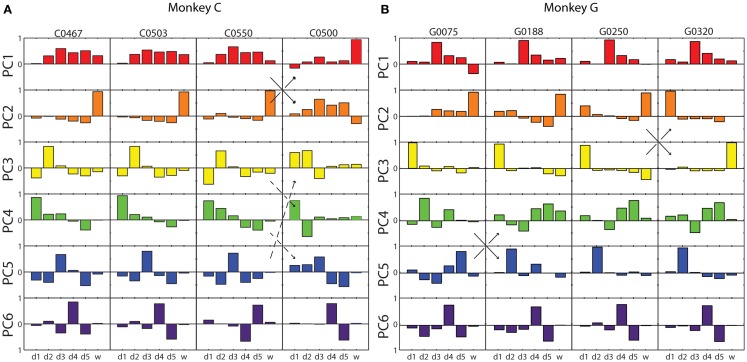
**Eigenvector components for each principal component in four illustrative sessions from each monkey**. **(A)** Monkey C; **(B)** Monkey G. Each of the eight columns displays the components of the six PC eigenvectors as a separate bar graph in each row, from PC1 (red) at the top to PC6 (purple) at the bottom, from a single session. Within each bar graph, the six bars represent the six components of the eigenvector projected onto each of the original six DoF axes, from d1 through w. Solid arrows indicate instances in which two similar eigenvectors swapped rank order reflecting that one accounted for somewhat more variance in one session, whereas the other accounted for more variance in the other session. Dashed arrows indicate an instance in which the composition of three eigenvectors changed between two sessions.

The six eigenvectors derived by PCA are illustrated for four selected sessions from each monkey in Figure 3. Within each frame, the eigenvector for a given PC (row) in a given session (column) is shown as a bar graph of its components along the original digit and wrist dimensions. In some cases, the patterns of correlated motion represented by a given PC changed rank order, indicating session to session differences in the relative amount of variance explained by the different patterns (black arrows in Figure 3). But on the whole, inspection of these data suggested considerable consistency from session to session and from monkey to monkey.

To examine the consistency of the patterns identified by PCA across all recording sessions more objectively, we performed average-linkage cluster analysis on all six eigenvectors from all sessions, using 1 minus the absolute value of the dot product between eigenvectors as a distance measure. Because the dot product between two unit vectors will be 1 if they point in the same direction and −1 if they point in exactly opposite directions, two eigenvectors that point along the same line in the six-dimensional space will have a distance measure of 0, and two eigenvectors that are orthogonal to one another (dot product of 0) will have a distance measure of 1.

**Figure 4:**
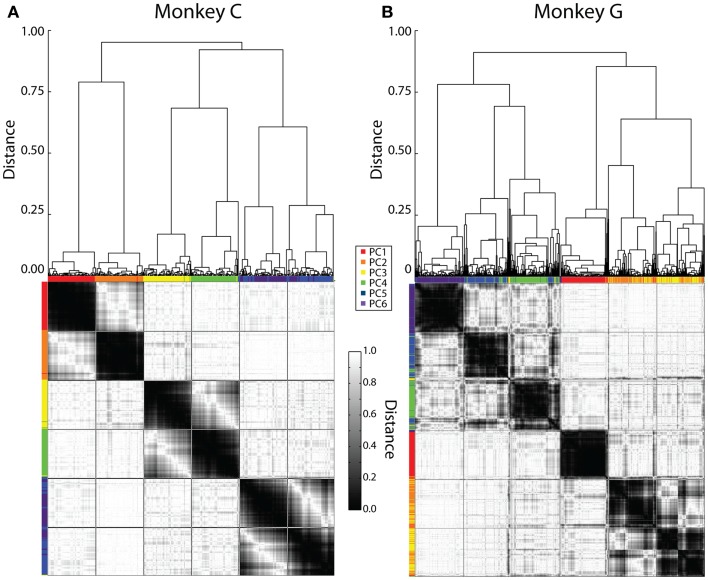
**Clustering of PC eigenvectors**. Shown here are the results of separate clustering for monkey C **(A)** and monkey G **(B)**. The resulting dendrogram is shown above and the distance matrix below. Colored ticks along the top and left sides of the distance matrix indicate the original PC rank-order from PC1 (red) to PC6 (purple) of each eigenvector represented by each column or row of the distance matrix. The distance matrix is symmetric about its main diagonal. The six major dark squares along this diagonal indicate that six relatively consistent kinematic synergies were present across sessions from both monkeys. Lines have been drawn on the distance matrix dividing both the rows and the columns into six groups of equal number.

Initially, this cluster analysis was performed on all the sessions from each monkey separately. Figure 4 illustrates the results, with a dendrogram above, a distance matrix below, and color bands along the margins of the distance matrix that show which rank-ordered PCs from different sessions were grouped together by the clustering process. Although our clustering method did not specify the number of groups expected, in each monkey six major groups of similar eigenvectors resulted, evident in the distance matrix as six dark regions of similar size along the main diagonal.

We therefore defined six kinematic synergies in each monkey by dividing the clustered eigenvectors into six groups of equal size, as illustrated by lines drawn on each distance matrix to create an evenly spaced, 6 × 6 square grid. If the eigenvectors had clustered into six perfectly distinct groups, with one eigenvector from each session in each group, then the six large dark regions along the main diagonal would have been perfectly delimited by these lines. While less than perfect, we felt that the borders of the dark regions were close enough to the squares delimited along the main diagonal for us to consider that the lines delimited six different kinematic synergies that were relatively consistent in each monkey. We refer to these six kinematic synergies as S1–S6.

**Figure 5:**
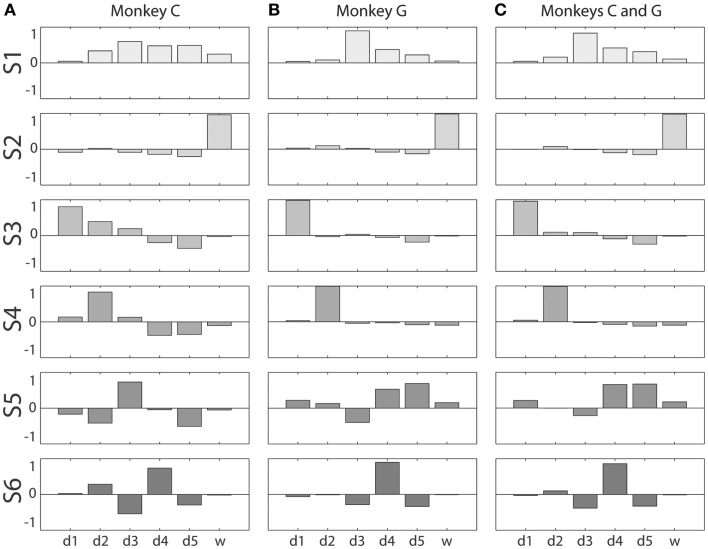
**Kinematic synergies**. Vector averaging of the eigenvectors clustered into each of the six squares along the main diagonal of the distance matrices of Figure 4 resulted in the six synergies – S1 through S6 – shown for monkeys C and G in **(A,B)**, respectively. **(C)** shows the six synergies resulting from the same process applied to the eigenvectors from all sessions from both monkeys together. Within each bargraph, the six bars represent the six components of that synergy’s eigenvector projected onto each of the six original DoF axes, from d1 through w.

To visualize each kinematic synergy, we vector-averaged all the eigenvectors assigned to a given synergy. Figures 5A,B show these averaged eigenvectors for each of the six kinematic synergies derived from the cluster analysis of the data from each monkey, C and G, respectively. In addition, we pooled the eigenvectors from the cluster analysis of both monkeys’ sessions and repeated the cluster analysis. Here, we again divided the distance matrix into an evenly spaced, 6 × 6 square grid (not illustrated), and vector-averaged the eigenvectors in each square along the main diagonal to define synergies for all sessions from both monkeys considered together. These average synergies across both monkeys are shown in Figure 5C.

The first synergy, S1, was characterized by motion of digits 3, 4, and 5 in the same direction, with d3 moving the most. In monkey C, S1 also included some motion of d2 and d6 in the same direction. S2 was dominated by movement of the wrist, d6. S3 was dominated by movement of the thumb, d1, with slight movement of d5 in the opposite direction. In monkey C, S3 also included some motion of d2 and d3 in the same direction as d1. S4 consisted primarily of motion of d2, in monkey C also including lesser motion of d4 and d5 in the opposite direction. S5 can be characterized as motion of d3 and d5 in opposite directions, in monkey G including motion of d4 in the same direction as d5. S6 comprises motion of d4 in one direction with motion of d3 and d5 in the opposite direction. The six average kinematic synergies found in the two monkeys thus were similar.

### Cross-correlation of M1 neuron firing rate and movement kinematics

**Figure 6:**
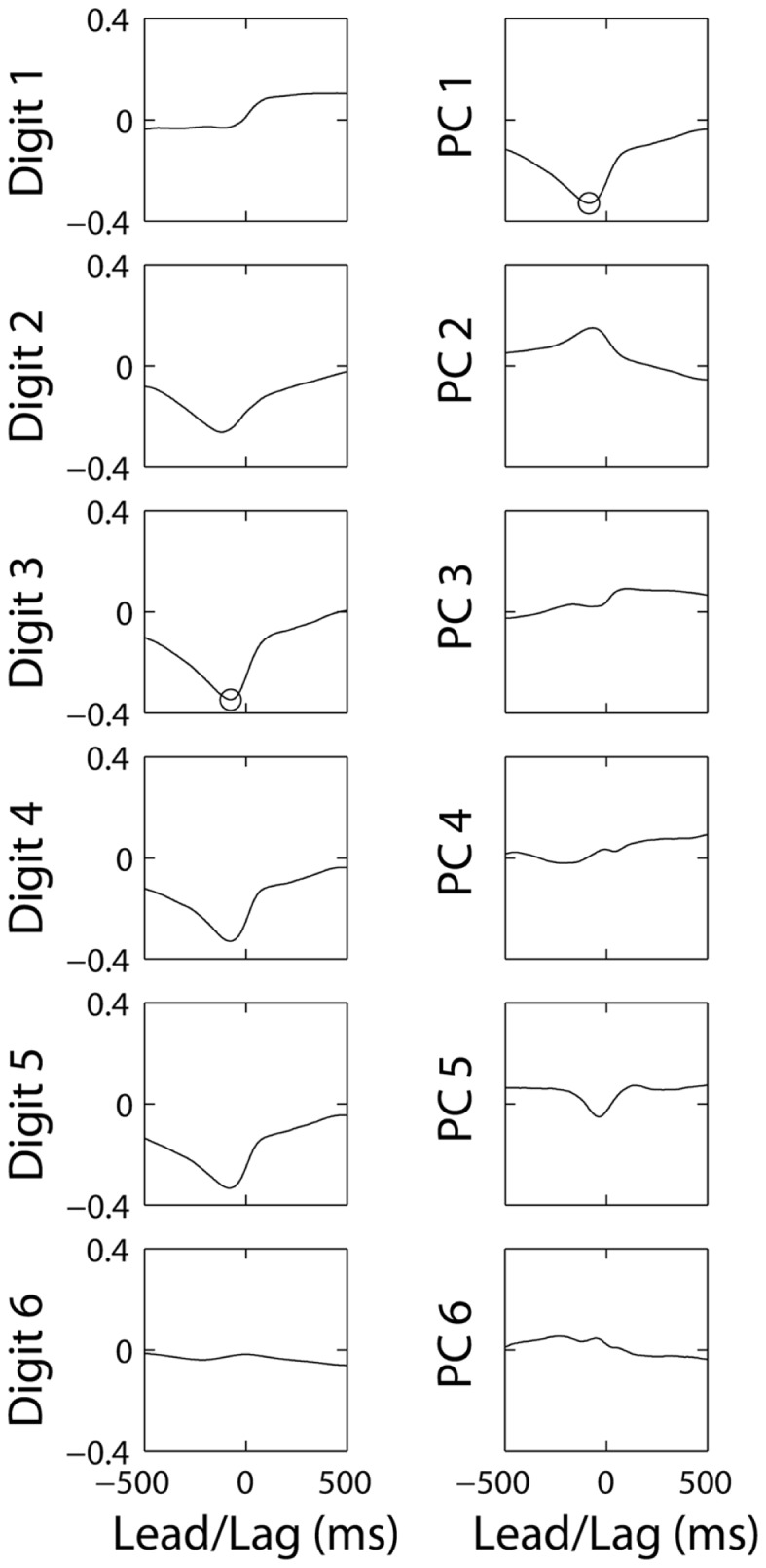
**Cross-correlations of the same M1 neuron’s firing rate with each original DoF and with each of the PCs from the same session**. The correlation coefficient (ordinate) is plotted as a function of the lead or lag (abscissa). Negative times represent those at which discharge of the neuron led the kinematic variable. Circles indicate the maximal absolute cross correlation for this neuron with any of the digits (Digit 3) and with any of the PCs (PC1).

For each M1 neuron, we preformed cross-correlation of its firing rate separately against the simultaneously recorded position of each digit and of the wrist, as well as against the temporal weighting of each of the six PCs derived from that simultaneous position data. Figure 6 shows the 12 resulting cross-correlation functions for neuron C0485, selected because it had relatively strong cross-correlations with finger kinematics. The cross-correlation functions with digits 1–6 in the left column show that this neuron correlated inversely with motion of digits 2, 3, 4, and 5, indicating that firing rate increased with extension of the digits. Because negative correlations here are just as meaningful as positive correlations, we focused on absolute values. The largest absolute value of any of these six cross-correlations (ρ = −0.34) occurred with d3 at a lead of −76 ms (indicated by the circle). The cross-correlations with the six PCs are shown in the right column. Here, the largest absolute value of any of the six cross-correlations (ρ = −0.32) occurred with PC1 at a lead of −84 ms (circle). The maximal absolute cross-correlation (MAXC) between the firing rate of this neuron and any of the digits thus was similar in both magnitude and timing to the MAXC obtained with any of the PCs.

### Comparing M1 neuron cross-correlations with original kinematics versus kinematic synergies

**Figure 7:**
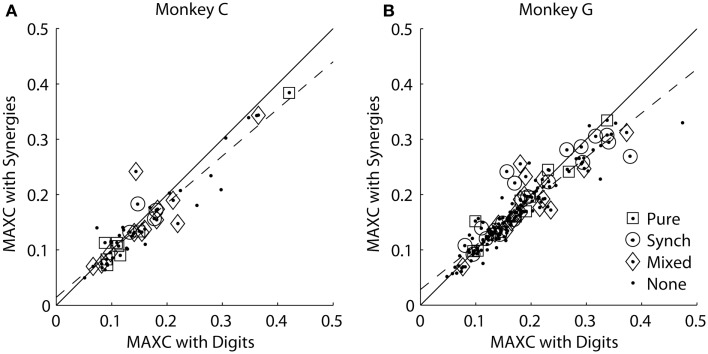
**Maximal absolute cross-correlations with original digit DoFs versus kinematic synergies in each monkey**. **(A)** Monkey C. **(B)** Monkey G. In each scatterplot, each point represents an M1 neuron plotted at the coordinates of its MAXC with any digit (abscissa) versus its MAXC with any of the average kinematic synergies from that monkey (ordinate). The solid line has a slope of 1.0, and the dashed line is the linear regression best fit to the data. Open symbols indicate the points representing neurons that had different types of effects in spike-triggered averages of EMG as indicated by the legend.

We reasoned that if an M1 neuron represented one of the kinematic synergies identified by PCA, then the cross-correlation of its firing rate with that synergy should be stronger than its cross-correlation with any of the individual digits or the wrist. For each monkey, we therefore plotted each M1 neuron’s MAXC with any of the digits against its MAXC with any of the average synergies. The resulting scatterplots are shown separately for the two monkeys in Figure 7. Here, values along the ordinate represent MAXC values obtained with the kinematic data projected along the six averaged eigenvectors shown in Figures 5A,B. Similar results were obtained, however, using the projection along the PC eigenvectors from each neuron’s individual recording session (as illustrated in Figure 3). Across the population of neurons from each monkey, MAXC values with the digits and with the synergies were correlated strongly with one another (using averaged synergies: monkey C, ρ = 0.94, *p* < 10^−22^; monkey G, ρ = 0.93, *p* < 10^−69^; using individual session PCs: monkey C, ρ = 0.94, *p* < 10^−22^; monkey G, ρ = 0.87, *p* < 10^−48^). Some points fell above the line of unity slope (solid line), indicating that for these neurons the MAXC with one of the synergies was greater than the MAXC with any of the digits. But paired testing showed that most points fell below the line of unity slope, indicating that for most M1 neurons the MAXC with one of the digits was greater than the MAXC with any of the synergies (synergies: monkey C, *z* = 3.01, *p* < 10^−2^; monkey G, *z* = 4.02, *p* < 10^−4^; PCs: monkey C, *z* = 1.99, *p* < 0.05; monkey G, *z* = 2.17, *p* < 0.05, Wilcoxon signed rank tests). Furthermore, in each monkey, points representing neurons with higher MAXC values tended to fall farther below the line of unity slope. The line best-fitting the data in each monkey (dashed line) had a slope significantly less than 1 (*p* < 0.05; synergies: monkey C, *m* = 0.85; monkey G, *m* = 0.80; PCs: monkey C, *m* = 0.84; monkey G, *m* = 0.74). Overall, rather than correlating more strongly with the kinematic synergies identified by PCA, the firing rates of M1 neurons, particularly those more strongly cross-correlated with finger and wrist kinematics, thus had somewhat stronger cross-correlations with the position of one of the digits or the wrist than with any of the average synergies or individual PCs.

Because some M1 neurons correlated most strongly with a kinematic synergy (points above the line of unity slope) whereas others correlated most strongly with an original DoF (points below the line), we also considered the possibility that the transformation from synergies to the muscle activation needed to drive them might occur at least in part within M1. More specifically, M1 neurons with relatively direct output to spinal motoneuron pools, particularly groups of cortico-motoneuronal (CM) cells with output to a similar subset of muscles, might produce patterns of activation in multiple muscles that would facilitate a given synergy (26, 27). If so, then those neurons that had stronger correlations with synergies might be those with relatively direct output to spinal motoneuron pools, whereas those neurons that had stronger correlations with an individual digit or the wrist might be less likely to have relatively direct outputs to muscles.

Each of the present neurons had been tested for such outputs with spike-triggered averaging of rectified EMG activity (24, 25). We classified the spike-triggered average (SpikeTA) effects of each neuron as being pure (consistent with direct, monosynaptic connections to motoneurons), synchrony (including synchronization with other neurons that had connections to the motoneuron pools), mixed (pure and synchrony effects in different muscles), or none. Open shapes in Figure 7 indicate which neurons had which type of SpikeTA effect. We observed no relationship between the presence or absence of any type of SpikeTA effect in M1 neurons and their correlations with synergies versus original DoFs.

**Figure 8:**
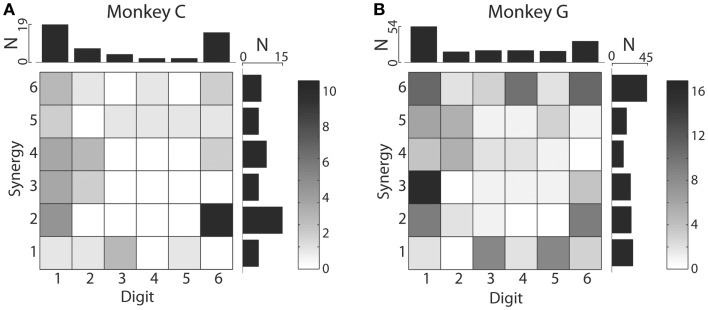
**Distributions of digits that cross-correlated most strongly with each original digit DoF and with each PC**. Two-dimensional histograms shown as grayscale matrices indicate the number of M1 neurons that had their MAXC with each joint DoF and with each PC in monkey C **(A)** and monkey G **(B)**. Note that the marginal histograms above shown that in both monkeys, the largest number of M1 neurons were best correlated with the thumb and then with the wrist, whereas the synergy with which the largest number of M1 neurons were best correlated differed between monkeys: S2 for monkey C, S6 for monkey G.

We also examined the distribution of MAXCs over the digits and kinematic synergies. The upper marginal histograms of Figure 8 show that in each monkey, the largest number of M1 neurons had their MAXC with d1, the thumb, and the next largest number with d6, the wrist. This is notable because in previous work the thumb and wrist have been found to exhibit higher degrees of independence than the other digits (22). The two monkeys did not show similar distributions of MAXCs across the synergies, however, as shown by the rightward marginal histograms in Figure 8. In monkey C, the largest number of neurons was best correlated with S2, which was dominated by d6, whereas in monkey G, the largest number of neurons was best correlated with S6, consisting primarily of motion in d4 with oppositely directed motion in d3 and d5 (Using individual session PCs, neurons in monkey C also were most often best correlated with PC2, and in monkey G with PC6.). Considering each neuron’s MAXC with a digit and its MAXC with a PC simultaneously, the two-dimensional histograms of Figure 8 show that in monkey C, the largest number of neurons was best correlated with d6 and S2 (dominated by d6 motion), and in monkey G the largest number was best correlated with d1 and S3 (dominated by d1 motion).

## Discussion

### Kinematic synergies of the hand in humans and monkeys

Previous studies have identified kinematic synergies of human hand motion by applying PCA to joint angles monitored during various activities, including grasping (1, 2, 11, 28), haptic exploration (4), activities of daily living (3), and finger spelling (29). PCA also has been applied to the kinematics of grasping in non-human primates (12, 14, 15). To our knowledge, none of the many other possible linear and non-linear mathematical methods for dimensionality reduction have been applied to hand kinematics. In preliminary studies, we examined the synergies identified in the present individuated finger and wrist movement task by independent component analysis (a linear method of dimensionality reduction that does not require the new basis to be orthogonal), but we found that for the present data the resulting independent components were not substantially different from the six original DoFs, i.e., the five individual digits and the wrist. For these reasons, the present study focused on the kinematic synergies identified with PCA. We found that these kinematic synergies were remarkably consistent across sessions and between monkeys. Nevertheless, we recognize that future studies using other approaches might better identify kinematic synergies used by the nervous system.

The studies cited above generally have found that: (i) a small number of the lowest order PCs account for a substantial majority of the variance in the motion of multiple joints; (ii) the synergies identified by PCA generally were similar from one subject to another; and (iii) the lowest order PCs represent a fundamental opening and closing of the hand involving similar motion in the thumb and all four fingers. In the present study, we likewise found that (i) the first PC accounted for ~50% of the variance, and the first three PCs for ~85%; (ii) the synergies identified by PCA were relatively consistent across sessions and between monkeys, and (iii) the first synergy (typically PC1) represented motion of the fingers in the same direction, albeit to different degrees in the two monkeys. In these three respects, the synergies identified here with PCA are similar to those identified in previous studies, although the present monkeys were instructed to move only one finger at a time insofar as possible.

We examined the structure of the kinematic synergies identified by PCA (Figure 5). Whereas S1 comprised simultaneous motion of the fingers all in the same direction, S2 in both monkeys consisted almost entirely of motion at the wrist, indicating that the wrist often moved relatively independently of the digits. S3 and S4, particularly in monkey G, likewise consisted almost entirely of motion of the thumb or of the index finger, respectively, indicating that each of these two digits also moved relatively independently. Compared to S3 and S4 in monkey G, S3 and S4 in monkey C included some motion of other radial digits (d1, d2, and/or d3) in the same direction, with motion of the ulnar digits (d4 and d5) in the opposite direction. In both monkeys, S5 and S6 represented simultaneous, oppositely directed motion in even closer subsets of the fingers. S5 comprised motion of the middle finger in one direction, with motion of other digits, most consistently the little finger, in the opposite direction. S6 comprised motion of the ring finger in one direction, with motion of the middle and little fingers in the opposite direction. In sum, whereas S1 comprised motion of multiple digits in the same direction, S2, S3, and S4 consisted of relatively independent motion of the wrist, thumb, and index finger, respectively, particularly in monkey G with some degree of radio-ulnar “contrast” (i.e., oppositely directed motion) in monkey C, and S5 and S6 consisted of increasingly close contrast among the more ulnar digits.

These features of the synergies identified with PCA may be related to findings on the relative independence of the digits and the structure of muscles in the macaque hand. Our previous studies of the individuated finger and wrist movement task performed by different monkeys demonstrated that the thumb, index finger, and wrist moved with more independence than the more ulnar digits – the middle, ring, and little fingers (22). Instructed movements of these ulnar digits generally involved motion of them all in the same direction (e.g., S1), with slightly more motion of the instructed digit, as might be created by the combination of S1 with one or more of the higher-order, “contrast” synergies. The combination of S1 and S6, for example, could produce more motion of the ring finger (d4) than other digits.

None of the kinematic synergies identified with PCA appeared to correspond to the activation of a particular muscle, however. Although S1 might be thought to reflect the action of the extrinsic multitendoned finger muscles – FDP, FDS, and EDC – in macaques FDP consists of two major compartments: FDPr, which exerts the most tension on d2, less on d3, and still less on d4; and FDPu, which exerts the most tension on d5 and d4 and less on d3 (30, 31). And the largest part of FDS acts on d3 and d4. Indeed, prior studies have indicated that flexion of each finger is produced by a different combination of activity in FDPr, FDPu, and FDS (32). S2 might be thought to reflect the action of muscles that act only across the wrist – FCR, FCU, ECR, and ECU – but ED23, EDC, and ED45, all are activated during wrist extension along with ECR and ECU. And higher-order synergies that include motion of some digits in one direction with motion of other digits in the opposite direction would have to be produced by coordination of forces acting on different digits in different directions. Few if any of the kinematic synergies identified by PCA, thus appear to represent the action of single muscles. Rather, each kinematic synergy is likely to involve coordinated activation in multiple muscles.

To some extent, the kinematic synergies identified here may reflect the particular mechanical constraints of the present individuated finger and wrist movement task (Figure 1) in which the instructed digit was required to move more than others, not only in flexion but also in extension. More ethologically natural human hand movements monitored during haptic exploration also showed synergies dominated by the motion of the thumb or the index finger, not unlike the present S3 and S4 (4) (S2 and S3 in their Figure 3). Although the same study showed that individuated movements of each digit could be reconstructed from the synergies identified, neither this nor other previous studies of kinematic synergies have elicited individuated movements of the middle, ring, and little fingers. Hence the “contrasts” between these digits represented by the present synergies S5 and S6, may not have appeared in more ethologically natural hand movements.

### Representation of kinematic synergies in the primary motor cortex

Overall, M1 neurons that had progressively stronger correlations with finger and wrist kinematics had stronger MAXCs with both synergies (or PCs) and original DoFs. If an M1 neuron specifically represented one of the kinematic synergies identified by PCA, then its firing rate would be expected to correlate more strongly with some synergy than with the motion of any of the individual digits or wrist. A minority of M1 neurons in each monkey – those represented by points lying above the line of unity slope in Figure 7 – in fact did show MAXCs with one of the synergies larger than with any of the individual digits or wrist. The majority of M1 neurons, however, showed a stronger correlation with the motion of an individual digit or the wrist than with any PC or kinematic synergy. Furthermore, in each monkey the M1 neurons that had progressively stronger correlations with kinematics showed particularly strong correlations with an original DoF rather than with a synergy. So although some M1 neurons might represent kinematic synergies in the present task, most M1 neurons represent these kinematic synergies no better than the original DoFs.

### Synergies and neural control of movement

Although we found little evidence that kinematic synergies are represented by M1 neurons more strongly than the original digit and wrist DoFs, our findings do not exclude a number of other possible ways in which synergies might be used by the CNS in controlling movement of the wrist, hand and fingers. First, methods other than PCA, either linear (e.g., independent component analysis), or non-linear (e.g., Isomap), may be necessary to identify kinematic synergies used by the nervous system. Second, although here we used digit and wrist positions as the original DoFs, synergies of other kinematic, and/or dynamic DoFs – such as velocity (15), acceleration, or force – might be represented more strongly in M1 neuron firing. Alternatively, rather than working in the domain of kinematic and/or dynamic synergies, the nervous system instead may control muscle synergies.

Much of the basic generation of such muscle synergies might occur at subcortical levels, including the PMRF and the spinal gray matter. Neurons in the intermediate zone of the lumbar spinal gray of the spinalized frog provide premotor drive for a limited number of muscular synergies (16), and rostral midbrain transection in the frog leaves most natural muscular synergies intact (19). These observations indicate that certain muscular synergies are mediated in the spinal cord. In monkeys, outputs from the PMRF produce relatively stereotyped facilitation of ipsilateral flexors and suppression of ipsilateral extensors (17, 33, 34), including hand muscles (35, 36), and PMRF neurons participate in visually targeted reaching movements (37, 38). Muscle synergies also have been identified during reach-to-grasp movements in both monkeys (20, 39) and humans (40–42), and remain largely unchanged after stroke damages the frontal cortex (18). Together, these studies suggest that in primates, the PMRF may generate important muscular synergies. M1 neurons, acting on subcortical centers, on spinal interneurons, and on the motoneuron pools themselves, then might sculpt the output to muscles so as to produce a wide variety of individuated movements (43).

## Conflict of Interest Statement

The authors declare that the research was conducted in the absence of any commercial or financial relationships that could be construed as a potential conflict of interest.
